# Implementation and Early Outcomes of Laboratory Proficiency Testing Program for the National Wastewater Surveillance System, United States, 2024

**DOI:** 10.3201/eid3213.251612

**Published:** 2026-08

**Authors:** H. Auguste Dutcher, Dagmara S. Antkiewicz, Adélaïde J. Roguet, Martin M. Shafer

**Affiliations:** Wisconsin State Laboratory of Hygiene, University of Wisconsin–Madison, Madison, Wisconsin, USA

**Keywords:** wastewater surveillance, proficiency testing, SARS-CoV-2, respiratory viruses, influenza, quality assurance, benchmarking, United States

## Abstract

Underlying the core goals of the Centers for Disease Control and Prevention National Wastewater Surveillance System (NWSS) in the United States is the need for robust and reliable pathogen data. The rapid build-out of the NWSS network and participation of many laboratories with varying expertise in environmental molecular methods has resulted in a lack of method standardization and poor data comparability. Proficiency testing is a powerful strategy to address this challenge. We describe preliminary outcomes from 2 proficiency testing rounds with 38 total participating laboratories, where reported respiratory virus concentrations from replicate samples spanned roughly 2 orders of magnitude; the program identified key opportunities for improvement. Although still in its infancy, this program proved an effective tool for quantifying variability across the network, offering preliminary insights on method performance, and improving data quality through laboratory support. Our results suggest that wastewater proficiency testing for disease surveillance will improve data quality and strengthen the NWSS.

Wastewater-based surveillance (WBS) for pathogens is increasingly recognized as a valuable complement to traditional clinical monitoring, serving as a leading indicator of increased disease transmission and providing less biased, cost-efficient community monitoring (*1*,*2*). Through the Centers for Disease Control and Prevention (CDC) National Wastewater Surveillance System (NWSS), the United States has established a network of laboratories reporting pathogen concentrations in wastewater, supporting public health decision-making (*3*,*4*). However, the rapid expansion of the network has outpaced the development of standardized protocols, resulting in substantial methodologic heterogeneity across laboratories (*3*,*5*).

With limited tools to assess the existing diverse methods used by laboratories with varied WBS experience, the comparability and utility of the data submitted to the NWSS program are compromised. Many potential sources of data variability in WBS are known, but only a handful of studies have systematically investigated their relative influence on pathogen recoveries (*2*,*6*–*9*). To date, very few interlaboratory comparisons have been conducted (*10*,*11*). Furthermore, many laboratories have insufficient resources for method optimization and operate with limited expertise and support.

A recent report from the National Academies of Sciences, Engineering, and Medicine emphasized the importance of cross-network data standardization for endurance of the network and pointed to proficiency testing (PT) as an avenue to improve data comparability (*12*). PT programs are a well-established mechanism in clinical and environmental testing for benchmarking performance (*13*–*16*), yet to our knowledge no such program exists for pathogens in wastewater, except for the Canada-focused program operated by the Ontario Clean Water Agency (*17*). Moreover, PT programs can provide insights on inter- and intra-laboratory variability, identify low- and high-performing protocols, and guide the establishment of networkwide uncertainty metrics to aid in data interpretation (*5*).

We describe preliminary outcomes of the first 2 rounds of a PT program designed and implemented by the Wisconsin Wastewater Monitoring Program (WWMP), a Center of Excellence in wastewater surveillance (WS) supported by the Centers for Disease Control and Prevention. We report on variability in pathogen quantification, methodologic diversity, and the effect of PT participation on laboratory performance. We identify key factors that contribute to interlaboratory variability, paving the way for future recommendations that could improve cross-network comparability. Finally, we discuss the implications of our findings for future rounds of our PT program as part of a broader effort to strengthen nationwide WS.

## Methods

We spiked influent from the same water reclamation facility with inactivated influenza A/B and respiratory syncytial virus and inactivated SARS-CoV-2 B.1.1.7 whole virus (Microbiologics, https://www.microbiologics.com) at levels above the detection limit (Appendix). We added spikes per tube to preserve material (round 1) or to a bulk sample prior to aliquoting (round 2). We evaluated wastewater samples for select physiochemical characteristics, which were consistent with historical norms. We prepared 3 replicate samples per participant by using a polypropylene churn splitter (SP Bel-Art, https://www.belart.com) and shipped all samples chilled for next-day delivery. We verified replicate consistency (sample homogeneity) with the start-to-finish turbidity (Appendix Table 2) and digital PCR analysis. We conducted a 2-week decay study to ensure stability of the viral signal over the assigned sample analysis window. Viral concentrations decreased an average of 30% at 1 week from preparation for influenza and SARS-CoV-2 (Appendix Table 1).

## Results and Discussion

The WWMP PT program maintained some elements of traditional PT while incorporating features designed to support laboratories and improve data comparability across the NWSS network. Participating laboratories received wastewater samples, and we instructed them to perform their standard wastewater pathogen protocols (consistent with NWSS, which imposes no prescribed workflow). Alongside quantification results, laboratories submitted detailed metadata of their wastewater processing workflows (https://doi.org/10.6084/m9.figshare.32301843. We used those metadata for quality assurance and to better understand the methodologic factors that can influence performance.

### Metadata Overview and Influence of Sample Pretreatment on Data Interpretation

**Figure 1:**
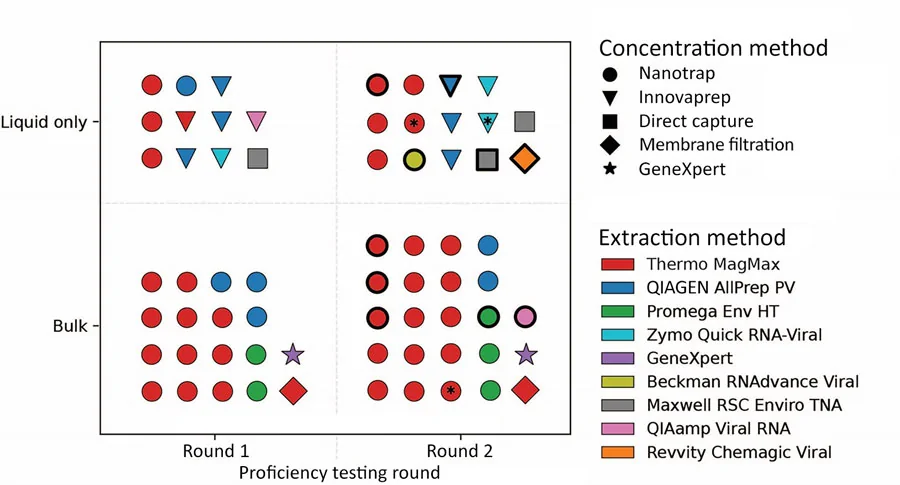
Laboratory workflows represented by the participants of the first 2 rounds of the Wisconsin Wastewater Monitoring Program wastewater proficiency testing program in study of implementation and early outcomes of laboratory proficiency testing program for National Wastewater Surveillance System, United States, 2024. Each symbol represents a single laboratory’s wastewater processing workflow (some laboratories tested multiple workflows). Laboratories are separated by the wastewater fraction processed (y-axis). Bold outline indicates new participants in round 2. Asterisks indicate laboratories that implemented a method change from round 1 to round 2. Nanotrap (Ceres Nanosciences, Inc., https://www.ceresnano.com); InnovaPrep (InnovaPrep LLC, https://www.innovaprep.com); Direct Capture (Promega, https://www.promega.com); GeneXpert (Cepheid, https://www.cepheid.com); Thermo MagMAX (Thermo Fisher Scientific, https://www.thermofisher.com); QIAGEN AllPrep Power Viral (QIAGEN, https://www.qiagen.com); Promega HT Env Kit (Promega, https://www.promega.com); Zymo Quick RNA-Viral (Zymo Research, https://www.zymoresearch.com); Beckman RNAdvance Viral (Beckman Coulter, Inc., https://www.beckman.com); Maxwell RSC Enviro TNA Kit (Promega, https://www.promega.com); QIAmp Viral (QIAGEN, https://www.qiagen.com); Revvity Chemagic Viral DNA/RNA 300 Kit (Revvity, Inc., https://www.revvity.com).

We conducted the first 2 rounds of our WS PT program in 2024 and focused on the respiratory pathogens SARS-CoV-2, influenza A virus, influenza B virus, and respiratory syncytial virus, along with any fecal markers and process recovery controls assessed by participating laboratories. Across both rounds (involving 38 unique laboratories total), 27 participants were state public health laboratories, representing most of the state jurisdictions reporting to NWSS. Enrolled laboratories used a wide variety of concentration and extraction methods (Figure 1; Appendix Tables 4–6).

To assess the effects of viral partitioning between the liquid phase and particulate matter on method performance, we asked laboratories to specify the wastewater phase processed in their workflows. Most laboratories analyzed a bulk sample (18 laboratories in round 1 and 24 in round 2), defined as a sample with no solids removal. Laboratories processing liquid-only samples (11 laboratories in round 1 and 14 in round 2) used a solids removal method such as centrifugation or filtration. Prior work has shown that SARS-CoV-2 and other viruses can associate strongly with solids and that sample prefractionation can substantially influence measured concentrations (*18*,*19*). NWSS uses a purely relative measure to interpret and visualize wastewater data, which does not account for this bulk versus liquid only distinction; however, the magnitude of partitioning between liquid and solid fractions can vary by pathogen and may result in systematically different pathogen recoveries. To investigate this question, we selected results from the most prevalent concentration method (Ceres Nanotrap, https://www.ceresnano.com) and compared data from laboratories that analyzed bulk and liquid fractions. We observed no statistically significant difference in SARS-CoV-2 concentration distributions between the 2 fractions (Appendix Figure 2), although substantial variability across laboratories limits our ability to statistically resolve particle-partitioning effects. In addition, our PT metadata review revealed that some laboratories also pretreat samples with proteases, detergents, or both to help mobilize viral particles into the liquid phase, introducing additional complexity into the interpretation of liquid only versus bulk categories. Because our current definitions of liquid-only versus bulk might not accurately reflect true viral partitioning behavior, we opted to pool results across sample fractions for the purposes of this analysis.

### Value of Follow-up and Summary Report for Participants

**Figure 2:**
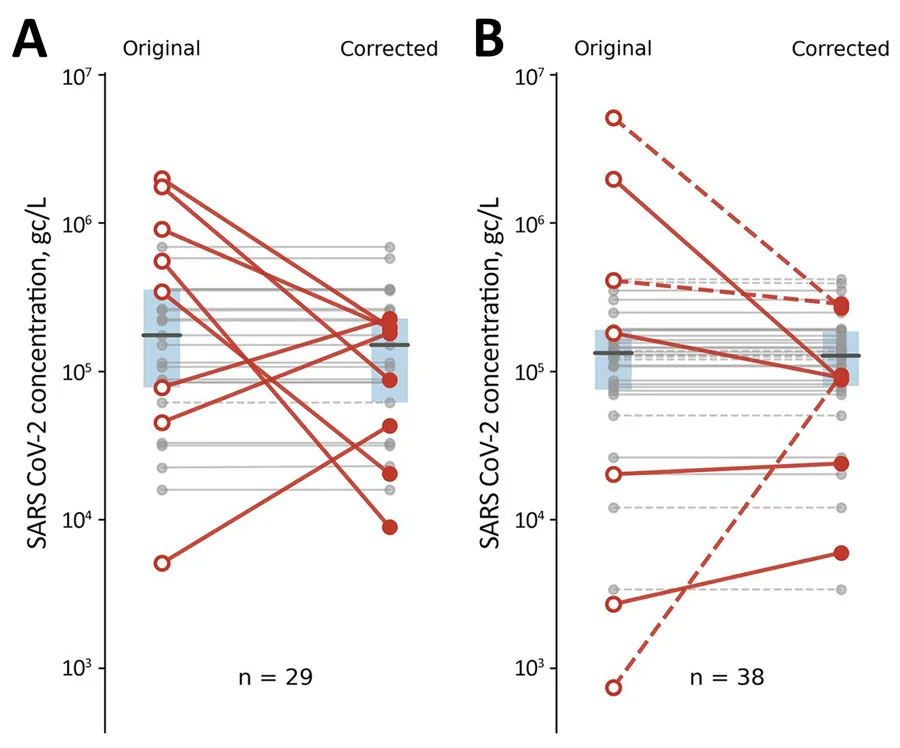
Reported SARS-CoV-2 concentrations in wastewater before and after Wisconsin Wastewater Monitoring Program proficiency testing (PT) intervention and laboratory resubmission of corrected concentrations in study of implementation and early outcomes of laboratory proficiency testing program for National Wastewater Surveillance System, United States, 2024 A) Round 1; B) round 2. Each point represents the reported concentration of SARS-CoV-2 from a given laboratory workflow, averaged across gene targets and replicates (n = 3). Total number per PT round is larger than the number of participating laboratories because of multiple workflows tested by some participants. Lines connect results from the same laboratory before and after correction; dotted lines indicate results from new participants in round 2. Red lines highlight results that required substantial correction reflecting issues such as unit conversion errors, misapplied processing factors, or other calculation discrepancies in the initially reported data identified during PT data review; no re-analysis of samples was performed. Blue shaded area denotes the interquartile region across laboratories before and after correction; line denotes median. gc/L, gene copies per liter.

As previously mentioned, our comprehensive data review and follow-up with participating laboratories was a key component of each PT round. Many laboratories benefited from the support provided by the WWMP throughout their participation, including receiving guidance that led to reporting corrections. In round 2, for example, 25 of 36 laboratories required follow-up for calculation, metadata, or other clarifications to ensure accurate reporting (Appendix Table 3). Some important corrections included unit conversion and dilution adjustments and correction of misapplied processing factors, which resulted in substantial changes (sometimes by orders of magnitude) to final pathogen concentrations (Figure 2). In the second round of PT, 3 of 9 new participants required corrections to their pathogen concentration calculations, compared with just 5 of 27 returning participants, highlighting the value of continued engagement.

After data analysis by the WWMP, we sent participating laboratories a report representing compiled data (Appendix Figure 8), including summary statistics for each pathogen and an associated z-score for each laboratory. That report enabled participants to independently assess their performance compared with all laboratories and those laboratories with similar workflows. Several survey respondents highlighted these benefits; one respondent noted, “It was very useful seeing our results plotted out with other labs to see how our results compare,” and another wrote, “Having this resource provides us with a clear way to verify that our processing methods are robust.” Overall, survey responses indicated that follow-up interactions and PT reports were valuable for participants and useful for cross-laboratory performance comparisons.

### Interlaboratory and Intralaboratory Variability

**Figure 3:**
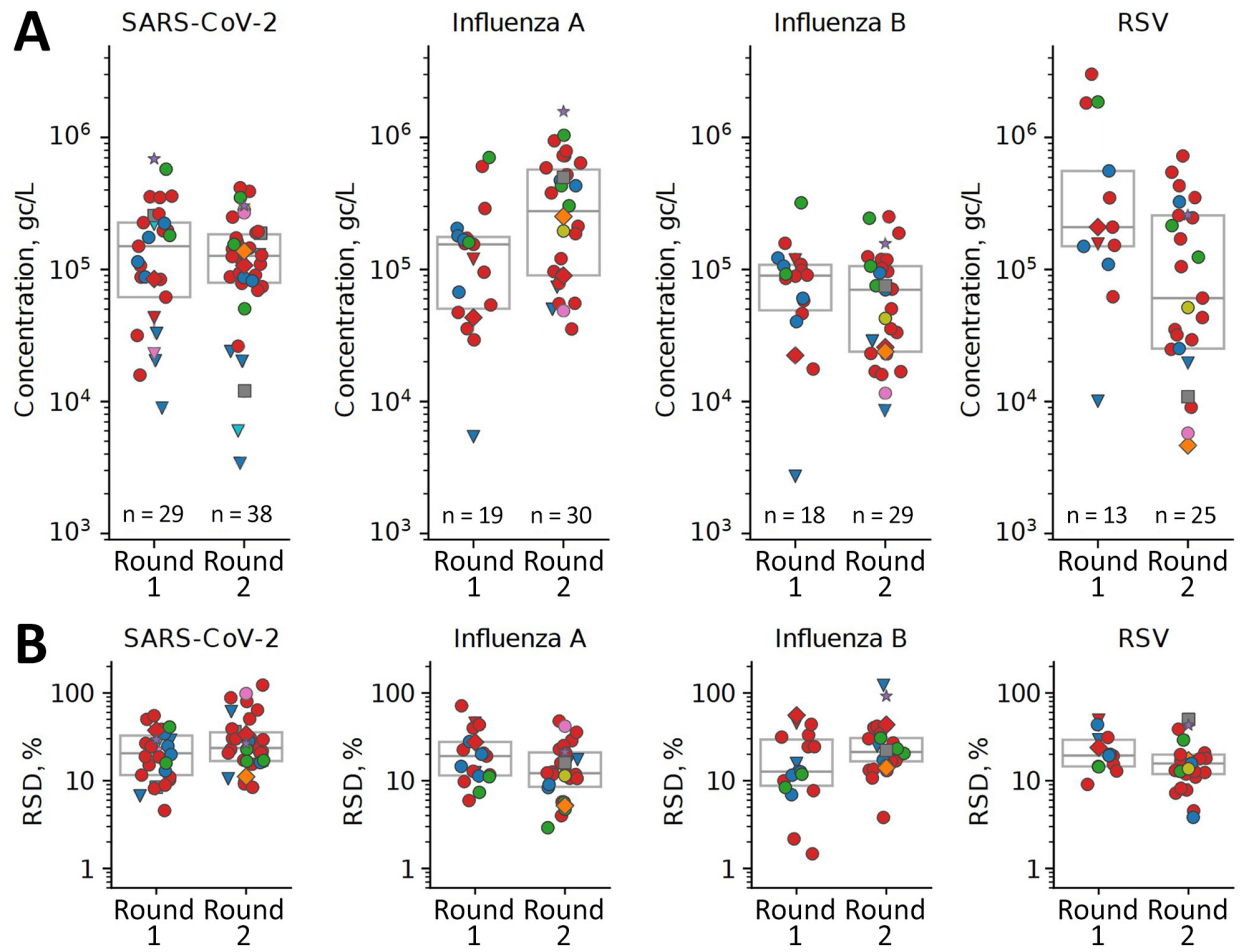
Results from first 2 rounds of Wisconsin Wastewater Monitoring Program proficiency testing, including respiratory pathogens SARS-CoV-2, influenza A, influenza B, and RSV in study of implementation and early outcomes of laboratory proficiency testing program for National Wastewater Surveillance System, United States, 2024. A) Concentrations of pathogens reported by the laboratories participating in proficiency testing rounds 1 and 2. Each symbol represents 1 laboratory’s results from a single workflow, averaged over gene targets (if applicable) and replicates (n = 3 for both rounds). B) RSD (%) of pathogen concentrations reported from the replicates in panel A (n = 3). Shapes and colors denote different methods of wastewater concentration and extraction (Figure 1). Horizontal lines within boxes indicate median, top lines of each box indicate 75th percentiles, and bottom lines represents 25th percentiles. gc/L, gene copies per liter; RSD, relative standard deviation; RSV, respiratory syncytial virus.

Compiled results showed that measured concentrations for each pathogen, which do not incorporate any recovery corrections (Appendix Figure 6), spanned approximately 2 orders of magnitude in each PT round (Figure 3, panel A), a modest improvement over the wide range reported by an interlaboratory comparison conducted in 2020 (*11*). The range observed in our exercises was comparable to 2 other interlaboratory comparison studies conducted with smaller laboratory cohorts (*17*,*20*). Normalization of pathogen concentrations by the fecal marker pepper mild mottle virus, which is not reported by all laboratories, did not consistently reduce data spread across pathogens (Appendix Figure 1, 3), and variances were not significantly different before versus after normalization, with the exception of SARS-CoV-2 in round 2 (p = 0.04 by Brown–Forsythe test).​ Restricting pathogen concentration comparisons to laboratories by using the most common workflow (bulk wastewater samples processed by using Ceres Nanotrap concentration and MagMAX extraction [Thermo Fisher Scientific, https://www.thermofisher.com]) modestly improved the comparability of reported results (range of 1.4 log_10_ units for SARS-CoV-2 in round 1, compared with nearly 2 log_10_ units for all laboratories [Appendix Figure 4]). However, sample size is limited given the overall diversity of methods used (Figure 1). Recruiting laboratories that all employ the same concentration and extraction method would enable a more rigorous assessment of cross-laboratory variability and could be a valuable design for future PT rounds.

To complement this cross-laboratory analysis, we also examined intralaboratory variation by using the 3 replicate samples included in both rounds of PT (Figure 3, panel B). Across both rounds and all pathogens, the interquartile range of relative SD across replicates was 12%–30%, and roughly 80% of all pathogen results had a relative SD <35%. Although no universally accepted benchmarks for intralaboratory variability exist for WBS, those values are very modest in the context of high interlaboratory variability and suggest that most laboratories can achieve a reasonably high level of reproducibility. Across rounds, SARS-CoV-2 z-scores were moderately correlated (Appendix Figure 7), particularly when laboratories that changed methods between round 1 and round 2 were omitted (adjusted r-squared = 0.669; p = 6.29 × 10^–7^), supporting NWSS’ use of site-relative reporting.

### Points of Intervention Identified in First 2 Rounds of PT

A comparative analysis of method performance from the 2 rounds of PT results is inherently limited by the sample size, specifically by the small number of results representing any 1 workflow. Nonetheless, preliminary results suggest some methods might be underperforming; for example, laboratories using the Innovaprep Concentrating Pipette (https://www.innovaprep.com) reported significantly lower pathogen concentrations than those using Ceres Nanotrap particles (p<0.01 by 2-sided Mann–Whitney U test) (Appendix Figure 5, panel A). When we compared raw viral concentrations (PCR instrument data reported in gene copies/µL of PCR reaction), that lower performance was statistically significant for SARS-CoV-2 and influenza B virus only (Appendix Figure 5, panel B). Even so, those preliminary results suggest that identifying underperforming methods can be successful within the structure of the program and may be a valuable route to improving cross-network comparability.

We also noted that, for SARS-CoV-2 (the only pathogen quantified by all participating laboratories), the lowest quartile contributed disproportionately to overall variability. In round 1, the lowest 25% of concentrations spanned approximately 0.8 log_10_ units, and in round 2, nearly 1.4 log_10_ units. In contrast, the upper 75% of values ranged just over 1 order of magnitude in round 1 and approximately 0.7 units in round 2 (Figure 3, panel A). That finding suggests that a relatively small subset of results accounts for a substantial fraction of interlaboratory variability. Focusing improvement efforts on laboratories and methods represented in the lowest recovery quartile could prove to be an effective means of improving network data comparability, at least for some pathogens.

### Qualitative Outcomes

Results from the follow-up surveys of participants highlight the PT program’s ability to identify problem areas and provide critical support to laboratories. Survey responses identified areas for program improvement and mirrored many of our existing plans for program expansion. For example, multiple laboratories suggested that a “PCR quantification only” PT round would be of interest. Narrowing PT exercises to focus on specific steps of the overall method may prove an important strategy to determine sources of variability, a strategy our program has since implemented with a digital PCR–focused round in late 2025. One respondent noted, “It is hard to compare our results to others since our protocol is not aligned with the majority. Maybe including an RNA sample for PCR only so we can determine if our PCR assay is comparable.” Another laboratory expressed interest specifically in high-consequence pathogens: “It would be interesting to investigate how different targets such as mpox and measles compare with the respiratory targets to determine if there are alternate processing methods needed to provide robust data for these targets.” Recognizing that need, our recently completed rounds of the PT program have included high-consequence pathogens that are relatively new additions to wastewater testing panels.

Of note, several laboratories reported correcting mistakes or reevaluating internal processes as a result of participation. In the follow-up survey, 4 respondents in round 1 and 5 respondents in round 2 indicated that their laboratories had corrected a mistake in calculations or their reported results (13 survey responses per round). One participant noted in their follow-up survey, “We found many problems with our method which without the PT report we would not have found.” Another laboratory reported changing methods to improve pathogen recovery after seeing their laboratory’s relative performance. Survey responses confirmed that enrolled laboratories welcome ongoing guidance; one respondent noted, “I’m looking forward to continued guidance on method optimization.” Together, those findings confirm that the program not only provides a comparative assessment of method performance but also is a powerful approach to improve data quality and laboratory practices.

## Conclusions and Future Directions

Early results from WWMP’s wastewater PT program demonstrated the value of structured interlaboratory comparisons in improving overall network data quality. Our findings corroborate previously reported variability in cross-laboratory pathogen quantification from wastewater and highlight the critical role of PT in identifying areas of improvement. From the first 2 rounds alone, we lack sufficient data to confidently reject any specific methods; instead, a key outcome of the initial rounds was identifying reporting errors, often stemming from calculation mistakes or unfamiliarity with standard practices. Incorporating individual consultations and collecting raw data (including metadata) as part of our program design proved essential for improving data quality. For multiple laboratories, the exercise provided an avenue for scientific guidance for their programs, and the act of participating alone improved data quality, suggesting that all laboratories reporting to NWSS would benefit from participating in PT.

Looking ahead, the goals for our wastewater PT program include expanding participation, incorporating additional pathogens, and improving various aspects of program implementation, such as metadata capture and reporting. Progress on those goals has already been made, including implementation of cloud-based data capture in round 3 and a measles-focused PCR quantification–only round 4. Contingent on continued financial support, we aim to continue our program implementation into the foreseeable future, with a long-term goal to make more specific recommendations regarding laboratory methodology and performance. Those efforts will improve the quality and comparability of WS data, strengthening NWSS and ensuring that the underlying data are as robust and reliable as possible for unbiased and defensible public health decision-making.

Appendix Additional information about implementation and early outcomes of laboratory proficiency testing program for National Wastewater Surveillance System, United States, 2024.
