# Supplementary material for: Implementation and Early Outcomes of Laboratory Proficiency Testing Program for the National Wastewater Surveillance System, United States, 2024
Source: Emerg Infect Dis. 2026 Aug;32(Suppl 1):S19–25. doi: 10.3201/eid3213.251612 (PMC13585686; doi:10.3201/eid3213.251612)
Supplement: Supplementary file 1 [file 25-1612-Techapp-s1.pdf]

Article DOI: https://doi.org/10.3201/eid3213.251612

EID cannot ensure accessibility for supplementary materials supplied by authors. Readers who have difficulty accessing supplementary content should contact the authors for assistance.

Implementation and Early Outcomes of Laboratory Proficiency Testing Program for National Wastewater Surveillance System, United States, 2024

Appendix

Additional Methods

Sample Characteristics

For Round 1 (WWS1) of wastewater PT we obtained 10 liters of 24-hour flow-proportional screened influent composite, collected from 12 am 05/22/2024 to 12 am 05/23/2024 at a large Wisconsin Water Reclamation Facility. Round 2 (WWS2) of PT was prepared using a 10L grab of screened influent sample, collected at 12:30 am on 12/12/2024 from the same large Wisconsin Water Reclamation Facility. Both samples were evaluated for a selection of physicochemical characteristics and all of the parameters were unremarkable, with the values for both samples within the range typical for a large Wisconsin wastewater treatment facility. Total suspended solids were of 313 and 242 mg/L for Rounds 1 and 2, respectively. BOD_5 was of 318 and 351 mg/L for Rounds 1 and 2, respectively. Finally, $CDOB_5$ was of 296 and 358 mg/L for Rounds 1 and 2, respectively.

Preparation of PT Samples

A minimum of three 45 mL replicate PT samples per each enrolled lab were prepared using a polypropylene churn splitter (SP Bel-Art). The sample splitting protocol and verification of replicate consistency and sample homogeneity, verified with the start-to-finish turbidity time series (Appendix Table 2), was modeled after (*1*). For Round 1, each replicate polypropylene conical tube was individually spiked with inactivated SARS-CoV-2, influenza A (IAV), influenza B (IBV) and RSV viruses (details provided in section on Viral Spikes below), while in

Round 2 only IAV, IBV & RSV were spiked (native SARS-CoV-2 level is wastewater were above limit of detection (LOD) in December) and the spike was added to the large volume of influent prior to splitting.

Spike concentrations were optimized such that majority of laboratory methods should detect all targets at above LOD. A dedicated 2-week sample stability study was also conducted (for WWS1 only, since the same spike materials, as well as the same sampling site were used in WWS2), spanning the recommended sample processing time window and ensuring that laboratories delayed in their sample processing would still obtain valid results (see Appendix Table 1 for details). We have also assessed whether sample processing delay influenced recovered viral concentrations in Round 1 but found no apparent relationship; however, the vast majority of participants analyzed PT samples within 2-4 days of shipment and this narrow range of processing times may have limited our ability to detect clear patterns. All samples were placed in ziplock bags for secondary containment and shipped the following day with a combination of frozen and refrigerated gel-ice packs in insulated shippers for next day delivery.

Viral Spikes

The inactivated whole virus spike-in materials were obtained from Microbiologics (St. Cloud, Minnesota, USA): Inactivated Influenza A/B and Respiratory Syncytial Virus (Cat. # HE0044N) and Inactivated SARS-CoV-2 B.1.1.7 Whole Virus (Cat. # HE0071N).

WWS1

A total of two Microbiologics HE0044N kits and two HE0071N kits (containing 5 pellets each) were added into 11 mL of 1X PBS and combined in one 50 mL conical tube. The virus suspension was then gently swirled and incubated at RT in a BSC for a minimum of 30 minutes, after which it was mixed again before adding 200 ul of the viral mix solution to each of the 50 ml conical tubes containing 45 mL of PT wastewater sample.

WWS2

A total of three Microbiologics HE0044N kits containing 5 pellets each were added into 15 mL of 1X PBS and combined in one 50 mL conical tube. The virus suspension was then gently swirled and incubated at RT in a BSC for a minimum of 30 minutes, after which it was mixed again before being added to the ~7L of raw influent sample placed in the churn splitter.

Reference

1. Chik AHS, Ho JJY, Srikanthan N, Dhiyebi H, Servos M. Design and validation of sample splitting protocol for comparison of SARS-CoV-2 quantification in wastewater. J Environ Eng. 2022;148:06022001. https://doi.org/10.1061/(ASCE)EE.1943-7870.0001994

Appendix Table 1. Average percentage of original concentration (week 0) of SARS-CoV-2, influenza A (IAV) and influenza B (IBV) at 1- and 2-week post-spike (WWS1). Note: all participants analyzed the PT samples within 1 week of receipt

Target Measured	SARS-CoV-2 N1 and N2 averaged		Influenza A		Influenza B	
Time-point	Week 1	Week 2	Week 1	Week 2	Week 1	Week 2
Replicate 1	49.33	32.54	61.96	32.07	57.78	37.25
Replicate 2	68.25	46.39	80.27	47.37	85.09	71.17
Replicate 3	84.81	70.34	77.44	38.25	78.92	54.78
Overall average	67.46	49.76	73.22	39.23	73.93	54.4

Appendix Table 2. Turbidity measurements time-series for both PT rounds

Sample name	Turbidity
WWS1 Start Rep 1	208
WWS1 Start Rep 2	206
WWS1 Start Rep 3	211
WWS1 Middle Rep 1	210
WWS1 Middle Rep 2	212
WWS1 Middle Rep 3	200
WWS1 End Rep 1	218
WWS1 End Rep 2	214
WWS1 End Rep 3	214
WWS1 Post-Aliquots Rep 1	211
WWS1 Post-Aliquots Rep 2	206
WWS1 Post-Aliquots Rep 3	215
WWS2 Start Rep 1	271
WWS2 Start Rep 2	268
WWS2 Start Rep 3	271
WWS2 Middle Rep 1	282
WWS2 Middle Rep 2	281
WWS2 Middle Rep 3	280
WWS2 End Rep 1	274
WWS2 End Rep 2	278
WWS2 End Rep 3	277
WWS2 Post-Aliquots Rep 1	268
WWS2 Post-Aliquots Rep 2	271
WWS2 Post-Aliquots Rep 3	280

Appendix Table 3. Summary of qualitative outcomes of WWS PT

Value	Round 1 (WWS1)	Round 2 (WWS2)
Number of labs contacted for clarification	14 of 29 (48%)	25 of 36 (69%)
Number of labs requiring corrections to final result calculations	11 of 29 (38%)	14 of 36 (39%)
Number of return participants requiring corrections to final result calculations	NA	5 of 27 (19%)
Number of labs with reported amounts not agreeing with PT calculations	12 of 29 (41%)	24 of 36 (67%)
Percent of survey participants who valued the PT exercise	100%	100%
Number of survey participants reporting correcting a calculation mistake	4 of 13 (31%)	5 of 13 (38%)
Number of survey participants re-evaluating their process based on PT results	1 of 13 (8%)	4 of 13 (31%)
Number of survey participants finding the PT report easy to understand	7 of 13 (54%)	12 of 13 (92%)
Number of survey participants satisfied with the support from WWMP	12 of 13 (92%)	100%

Appendix Table 4. Number of laboratories participating across both PT Rounds 1 (WWS1) and 2 (WWS2)

Category	WWS1	WWS2
Academic laboratories	2 (7%)	5 (14%)
Public health laboratories		
City	4 (14%)	5 (14%)
County	1 (3%)	1 (3%)
State	22 (76%)	25 (69%)

Appendix Table 5. Number of laboratories testing for spike-in BCoV, BRSV, MHV, or OC43 recovery controls across both PT Rounds 1 (WWS1) and 2 (WWS2)

Virus	WWS1	WWS2
BCoV	19 (83%)	19 (73%)
BRSV	1 (4%)	2 (8%)
MHV	3 (13%)	3 (12%)
OC43		2 (8%)

Bovine coronavirus virus (BCoV), bovine respiratory syncytial virus (BRSV), murine hepatitis virus (MHV), human coronavirus OC43 (OC43).

Appendix Table 6. Number of laboratories employing different quantification platforms across both PT Rounds 1 (WWS1) and 2 (WWS2)

Platform	WWS1	WWS2
ddPCR BioRad	6 (21%)	7 (19%)
dPCR Qiagen	22 (76%)	26 (72%)
dPCR Thermo		1 (3%)
GeneXpert	1 (3%)	1 (3%)
qPCR Thermo		1 (3%)

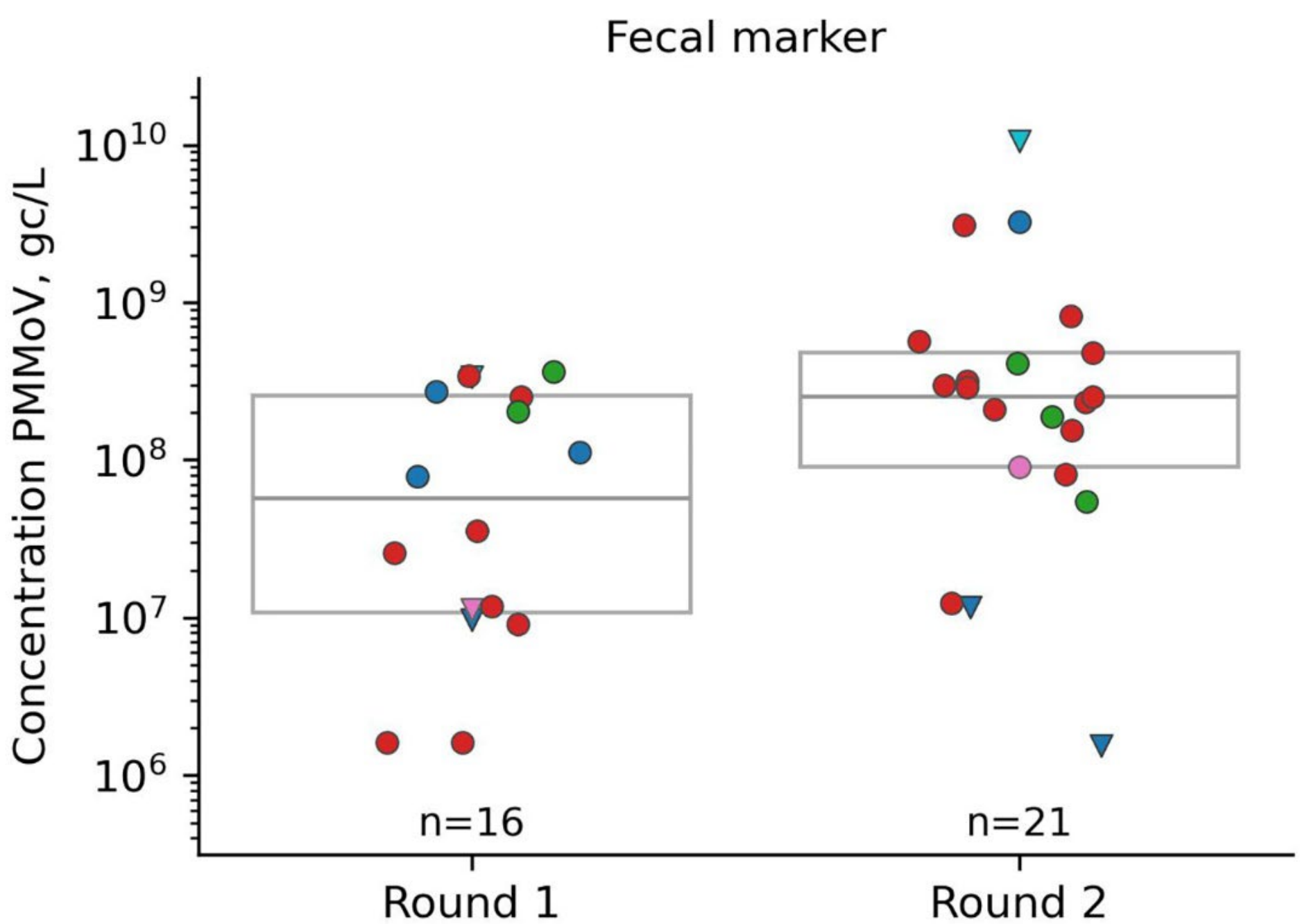

Appendix Figure 1. Concentrations of Pepper Mild Mottle Virus (PMMoV) in gene copies per liter (gc/L) of wastewater, reported by the laboratories participating in PT Rounds 1 (WWS1) and 2 (WWS2). Each symbol represents one laboratory's results from a single workflow, averaged over replicates (n = 3 for both rounds). Shapes and colors denote different methods of wastewater concentration and extraction, as described in Figure 1 or Appendix Figure 8 legends.

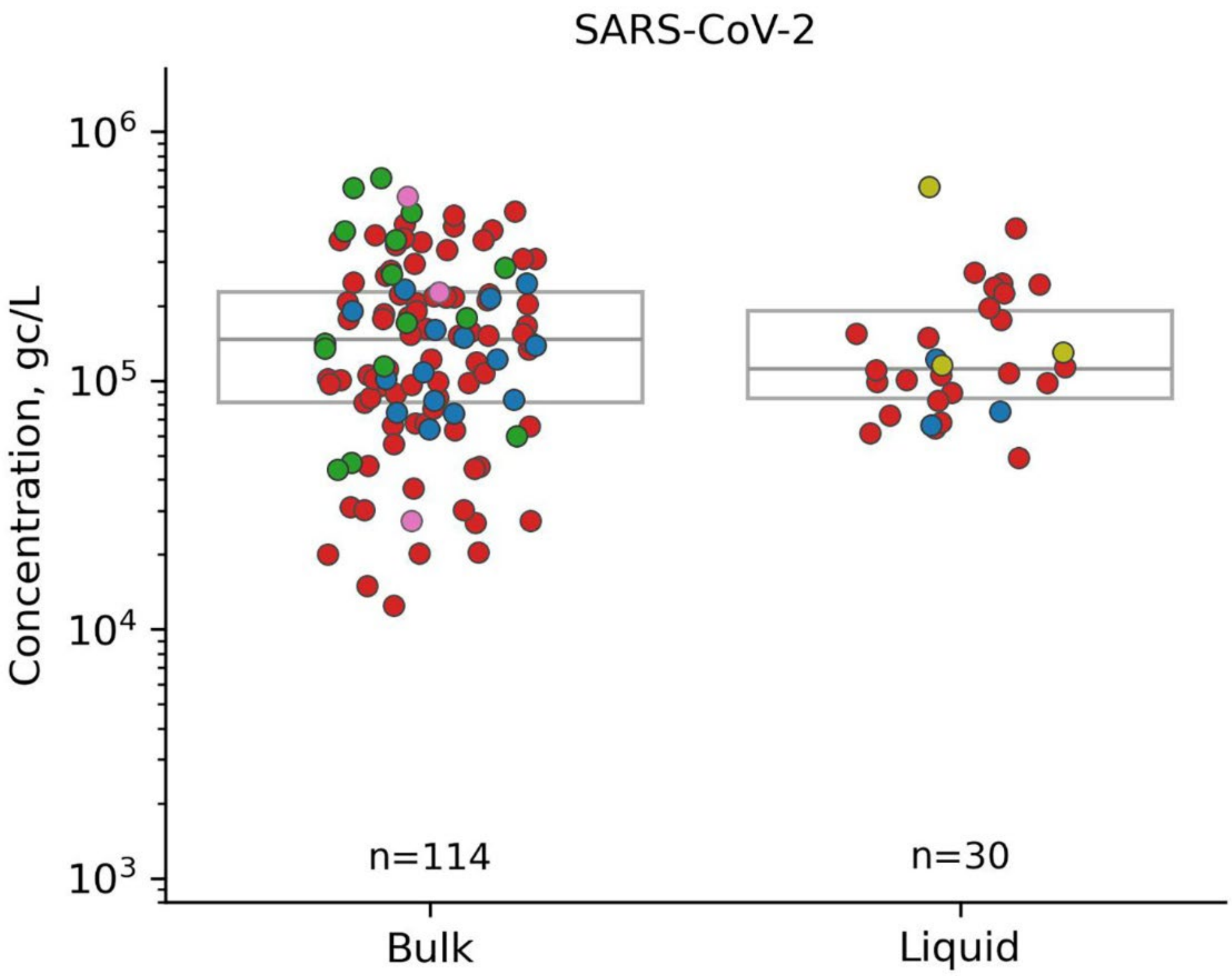

Appendix Figure 2. Concentrations of SARS-CoV-2 in gene copies per liter (gc/L) of wastewater, presented by fraction processed, from laboratories using Nanotrap concentration. Each point represents results from an individual sample (n = 3 for each lab), with results aggregated across PT rounds (WWS1 and WWS2). There is no significant difference in the distribution of results between bulk and liquid-only fractions, as assessed by two-sided Mann-Whitney U (SciPy v. 1.15.3).

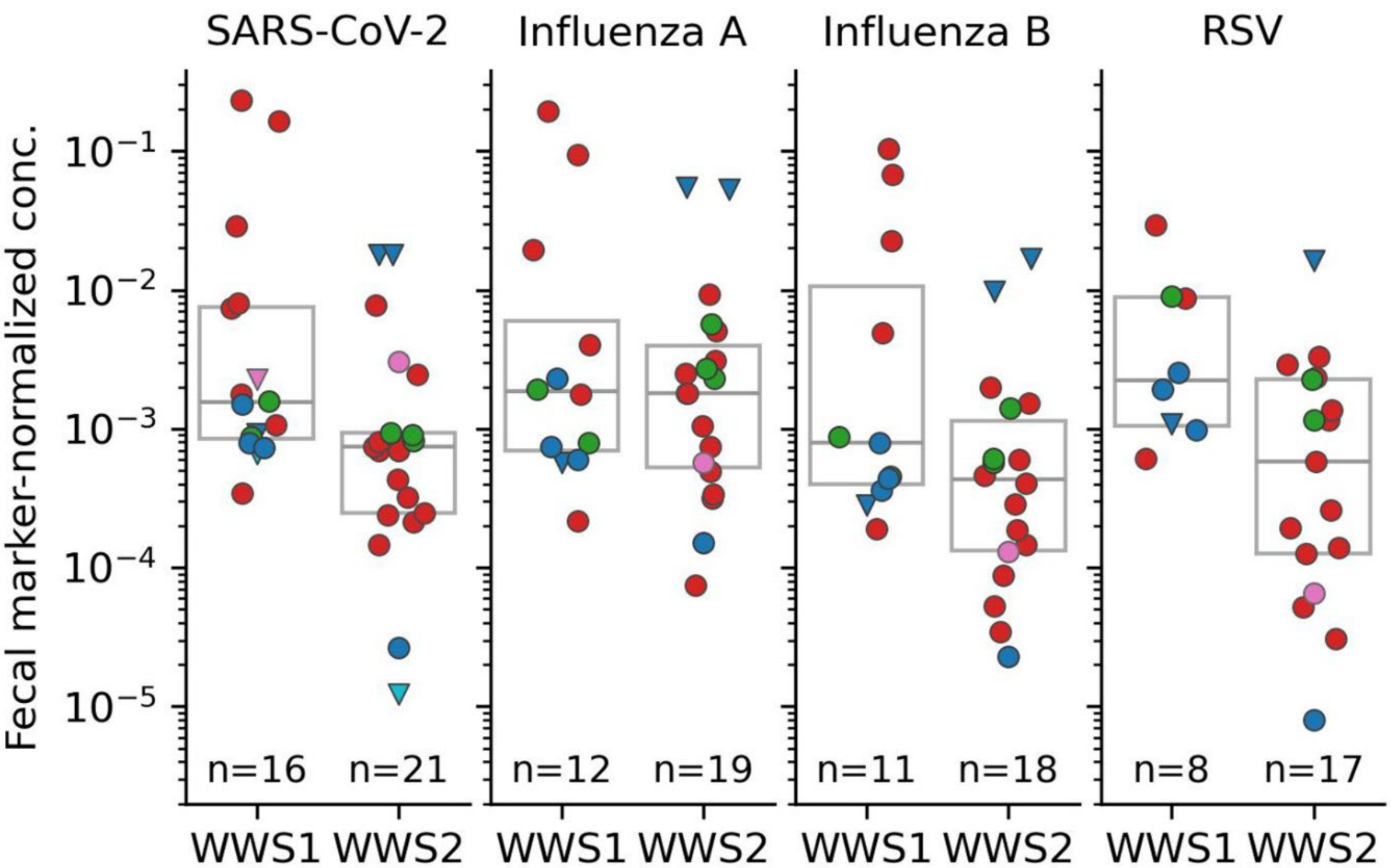

Appendix Figure 3. Concentrations of four respiratory pathogens (in gene copies per liter (gc/L) of wastewater) reported by the laboratories participating in PT Rounds 1 (WWS1) and 2 (WWS2), normalized to the concentration of fecal marker (PMMoV). Normalization by PMMoV did not consistently reduce data dispersion across pathogens. Variances were not significantly different before versus after normalization ($p > 0.1$), with the exception of SARS CoV-2 in Round 2 ($p = 0.04$, Brown Forsythe test).

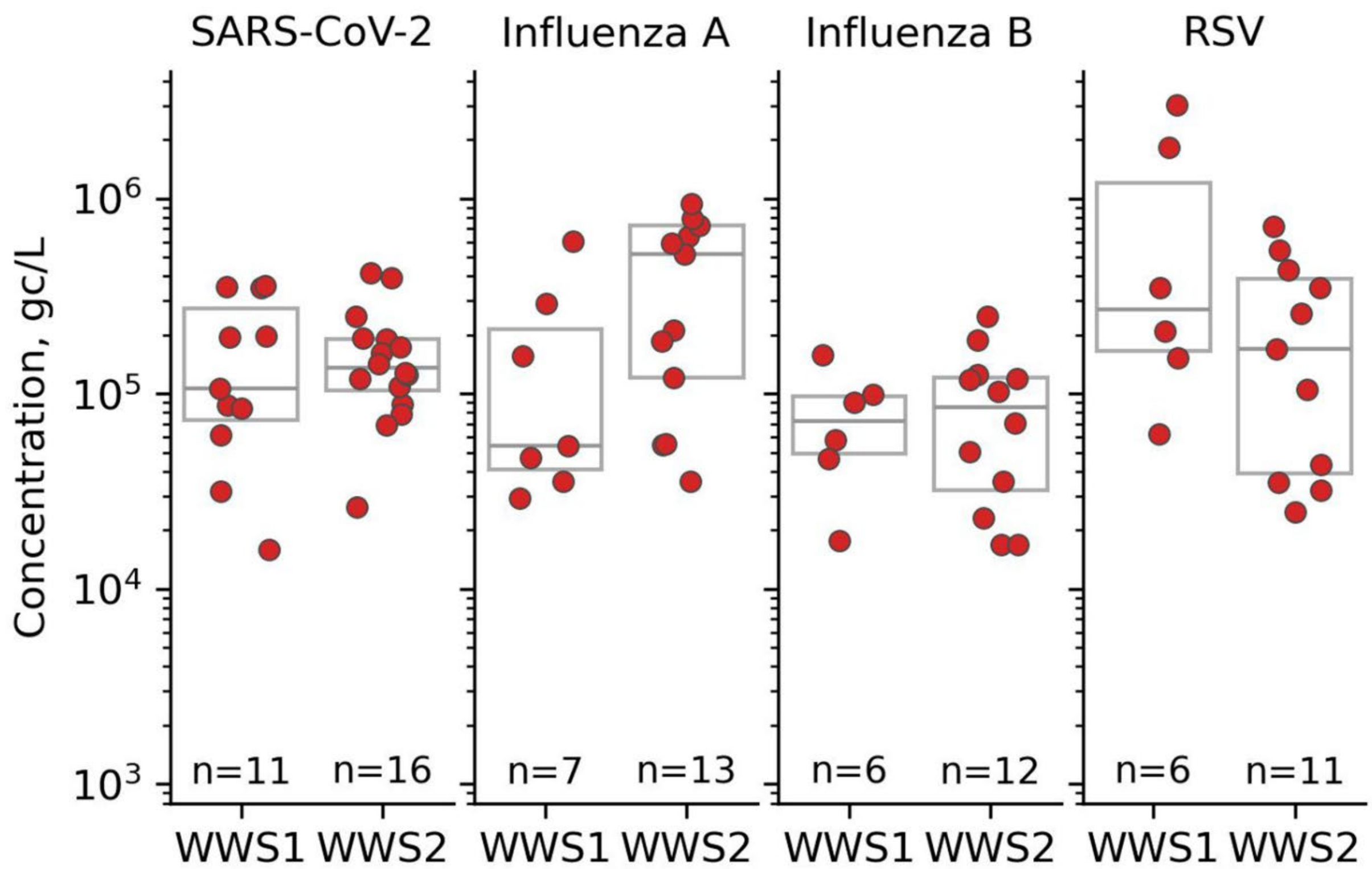

Appendix Figure 4. Concentrations of pathogens (in gene copies per liter (gc/L) of wastewater) from laboratories using Nanotrap concentration and Thermo MagMax extraction. Restricting analysis to a single concentration/extraction method only modestly reduces data set variability.

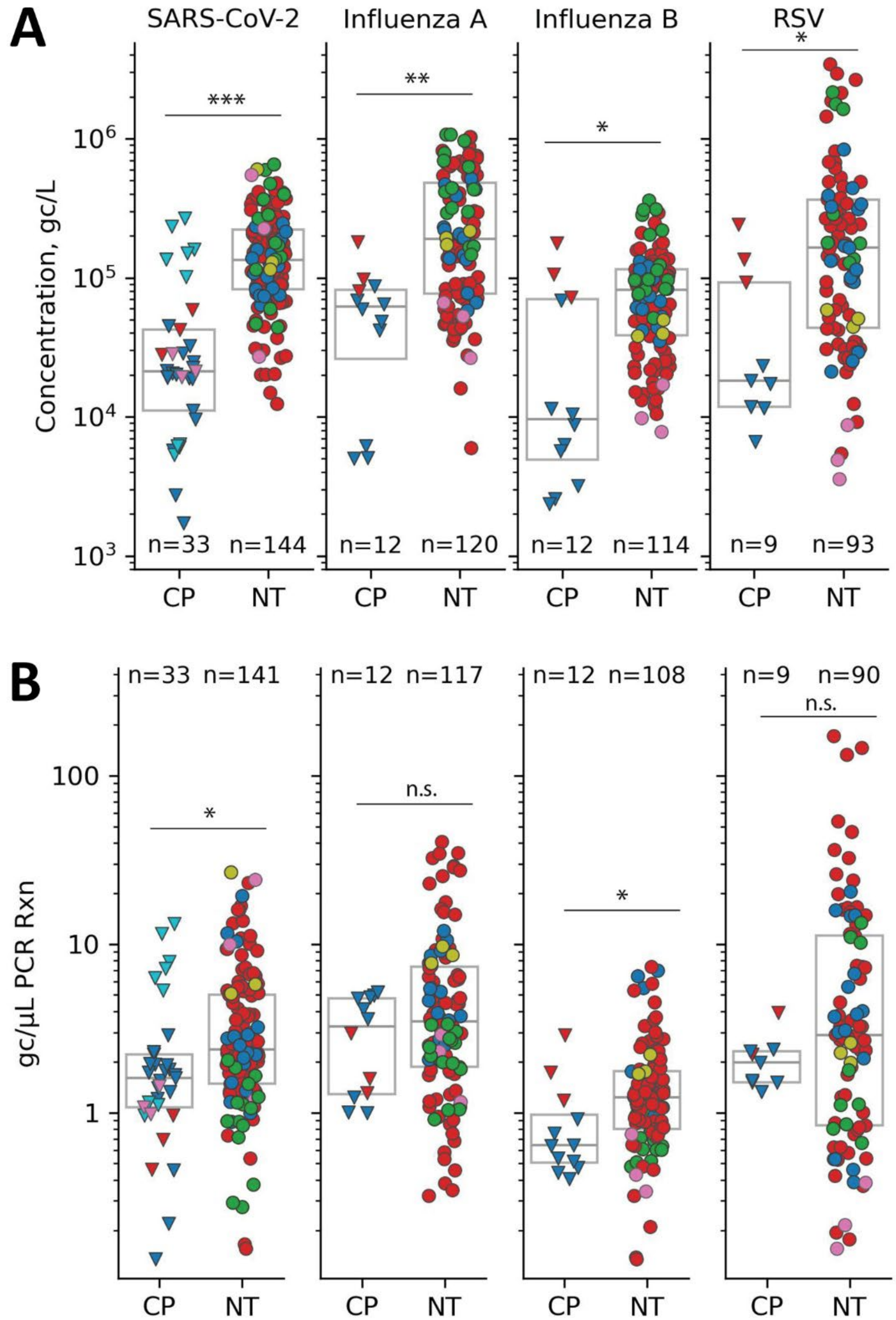

Appendix Figure 5. Concentrations of pathogens (A) in gene copies per liter (gc/L) of wastewater and (B) gene copies per microliter PCR reaction, by concentration method used (Innovaprep Concentrating Pipette, CP or Nanotrap, NT). (B) Each point represents results from an individual sample (n = 3 for each lab), with results aggregated across PT rounds (WWS1 and WWS2). *** = p < 1e-10; ** = p < 0.001; * = p < 0.01, n.s.: not significant; two-sided Mann-Whitney U (SciPy v. 1.15.3).

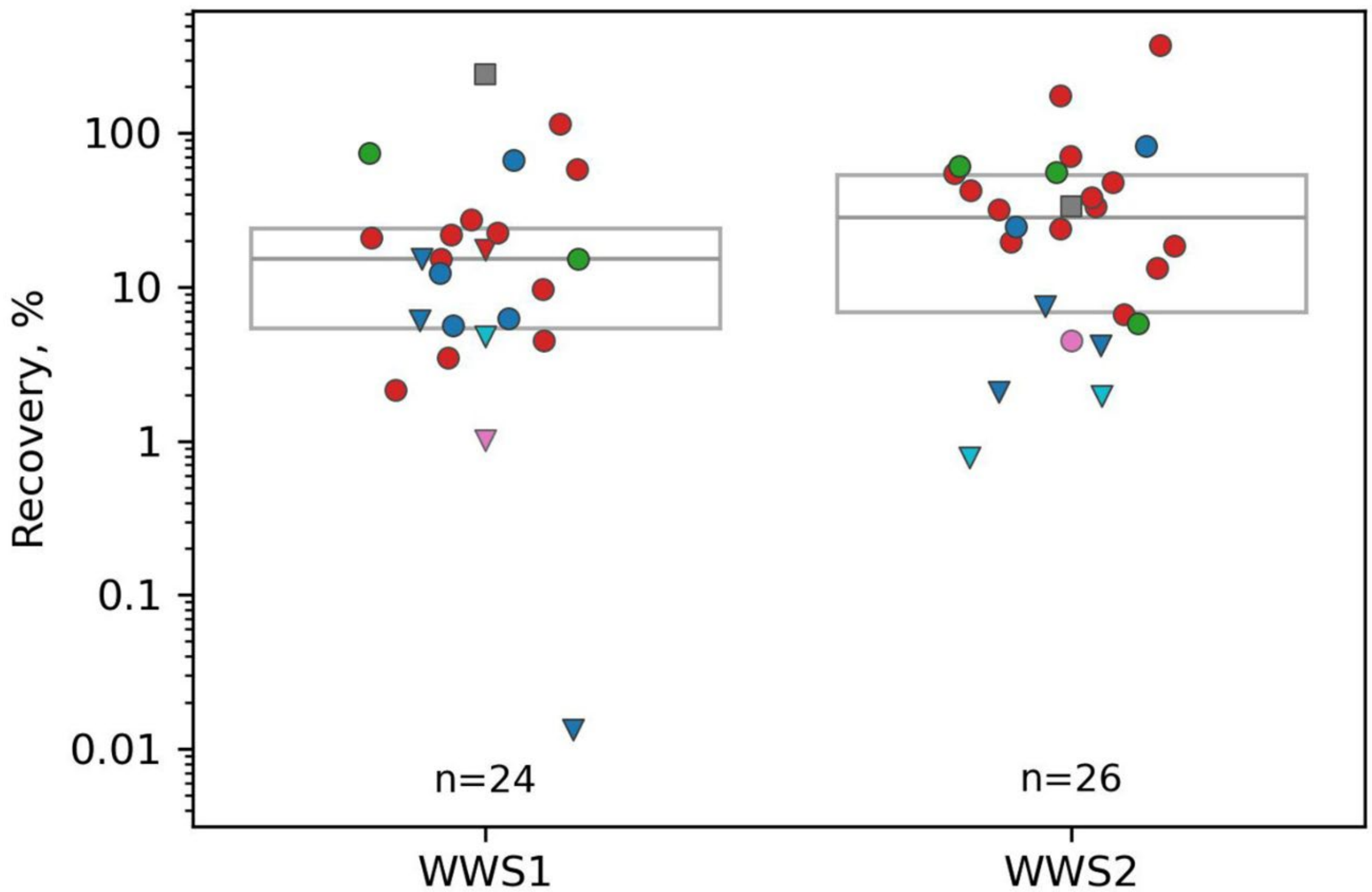

Appendix Figure 6. Percent recovery of process control, average across replicates (n=3).

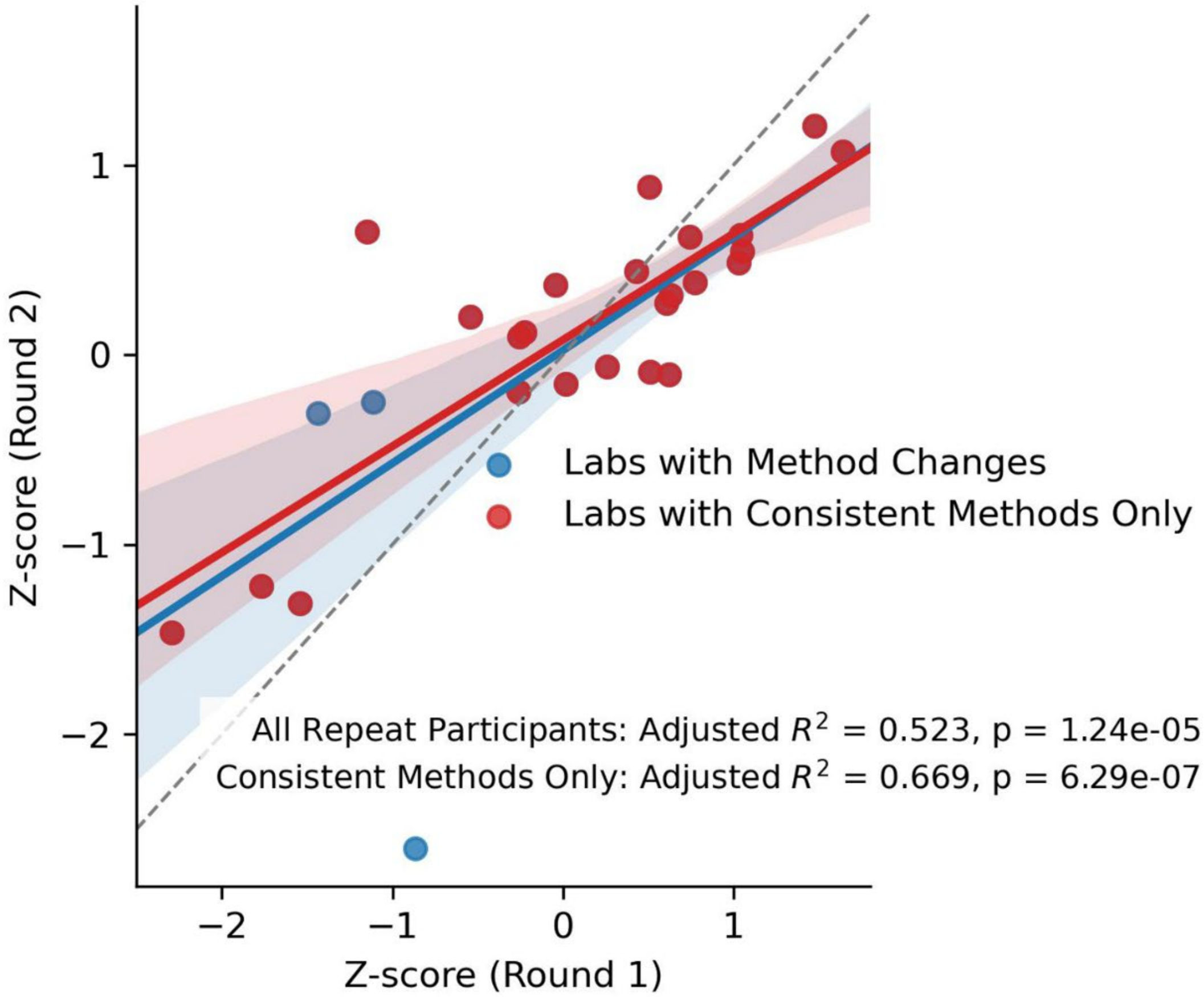

Appendix Figure 7. Z-scores associated with SARS-CoV-2 concentrations for laboratories that participated in both Round 1 and Round 2 of PT. Red dots highlight laboratories with consistent methods across rounds, whereas blue dots represent labs with different reported methods in Round 1 and Round 2, respectively. The lines show ordinary least squares regression (statsmodels version 0.14.5 package for python3); shaded area denotes the 95% confidence interval.

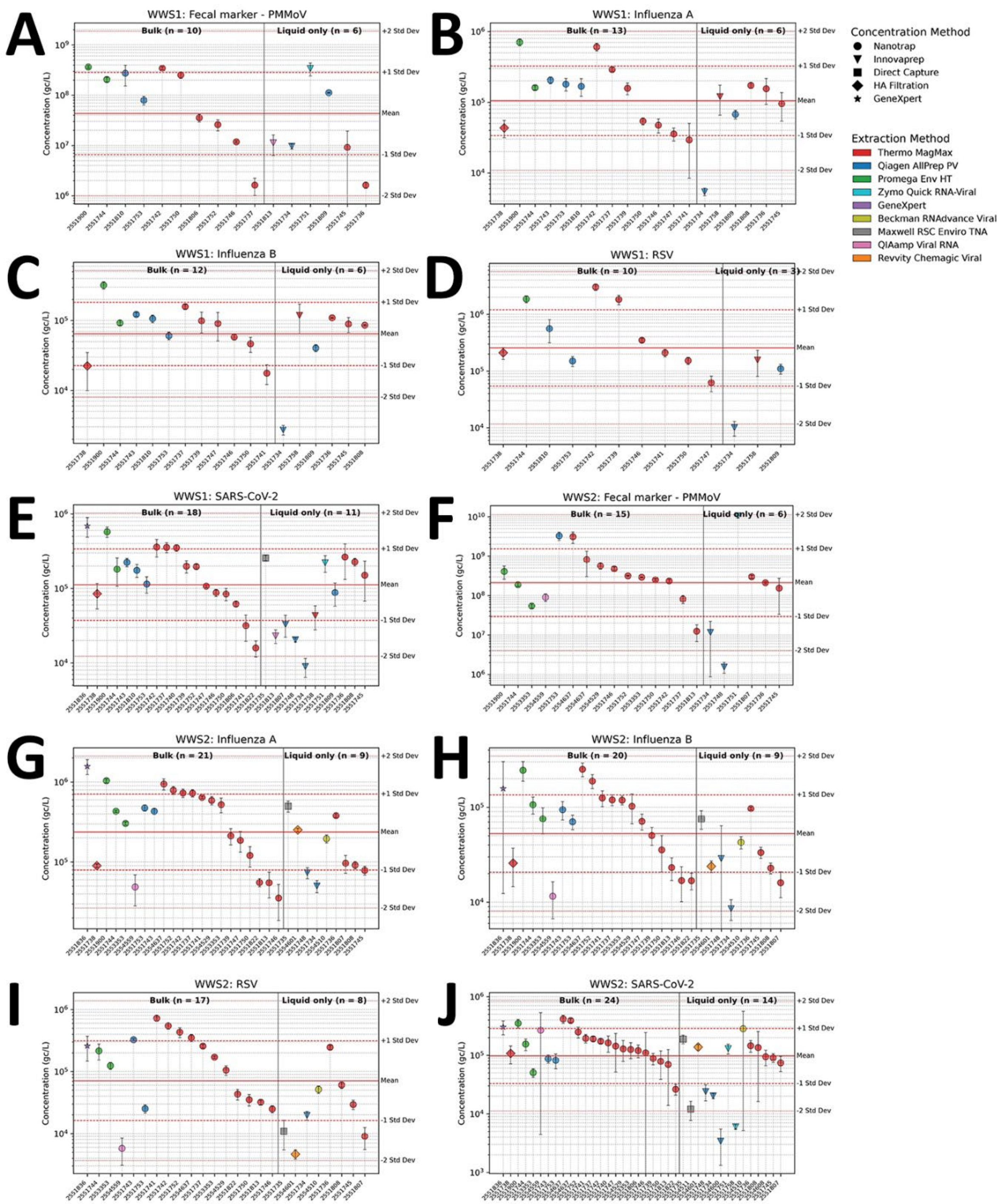

Appendix Figure 8. Summary reports for PT Rounds one (WWS1) and two (WWS2). Each plot shows concentrations of a given pathogen or a control target in gene copies per liter (gc/L) of wastewater. Results per laboratory are averaged across targets and replicates, with points representing the arithmetic mean and error bars standard deviation of three replicate wastewater samples. Red lines denote mean and standard deviation of results across all laboratories in log space. Concentration and extraction methods denoted according to the legend.
